# Interactions between the intestinal microbiome and host genes in regulating vibriosis resistance in *Cynoglossus semilaevis*

**DOI:** 10.3389/fimmu.2025.1644885

**Published:** 2025-09-03

**Authors:** Weiwei Zheng, Yadong Chen, Tao Yang, Zhihong Liu, Dong Xu, Huizong Han, Yaning Wang, Xiaoqing Xi, Tengteng Wang, Songlin Chen

**Affiliations:** 1 State Key Laboratory of Mariculture Biobreeding and Sustainable Goods, Yellow Sea Fisheries Research Institute, Chinese Academy of Fishery Sciences, Qingdao, Shandong, China; 2 Shandong Marine Resource and Environment Research Institute, Yantai, Shandong, China; 3 Rongcheng Marine Economic Development Center, Weihai, Shandong, China; 4 Laboratory for Marine Fisheries Science and Food Production Processes, Qingdao Marine Science and Technology Center, Qingdao, Shandong, China

**Keywords:** *Cynoglossus semilaevis*, intestinal microbiome, host genes, interactions, vibriosis resistance

## Abstract

**Background:**

Chinese tongue sole (*Cynoglossus semilaevis*) is now a commercially important flatfish species widely farmed in China. In recent years, frequent outbreak of vibriosis has caused high mortality rates and enormous economic losses to the aquaculture industry of Chinese tongue sole. The intestinal microbiome plays a crucial role in host immunity and protection against pathogen invasion. However, the interactions between the intestinal microbiome and host genes in vibriosis remain poorly understood.

**Methods:**

In this study, we investigated the changes in intestinal histopathology, intestinal microbiome and host gene expression in resistant and susceptible individuals at 7 days post infection with *Vibrio harveyi*, and identified the host gene-microbe correlations. Histopathological sections were conducted to detected the histopathological changes in the posterior intestinal tissues of resistant and susceptible individuals. 16S rRNA sequencing was performed to characterize the changes in intestinal microbial community. RNA-seq was used for the identification of host differentially expressed genes (DEGs). The associations between intestinal microbes and host genes were illustrated by perform an integrated analysis of the differential intestinal microbes and host DEGs.

**Results:**

Obvious histopathological differences were observed between the resistant and susceptible groups in terms of inflammatory cells infiltration, and tissue dissociation of mucosal layer. 16S rRNA sequencing analysis indicated that *Vibrio* increased but *Stenotrophomonas*, *Chryseobacterium*, *Delftia*, and *Salinivibrio* decreased in the susceptible group. Compared to the control group, 1,986 differentially expressed genes (DEGs) were detected in the susceptible group, significantly more than the 310 DEGs found in the resistant group. DEGs in the susceptible group were significantly enriched in immune-related GO terms, such as antigen processing and presentation, MHC protein complex, and immune response, and pathways, including antigen processing and presentation, phagosome, and proteasome. Through an integrative analysis of differential intestinal microbes and host DEGs, 207 strong gene-microbe correlations were identified.

**Conclusions:**

The results of this study indicate that *V. harveyi* infection severely damages the intestinal tissue and substantially alters the composition of intestinal microbiome and the expression of host genes, especially in susceptible individuals. Strong gene-microbe correlations may imply that the intestinal microbiome may interact with host genes to collectively regulate the vibriosis resistance in Chinese tongue sole, but the causality between them remains unestablished and requires further validation.

## 1 Introduction

Chinese tongue sole (*Cynoglossus semilaevis*, FishBase ID:17172), naturally distributed in the coastal areas of China, Japan, and Korea, is now an economically important mariculture flatfish species in China due to its favorable taste and high nutritional value (1, 2). As one of the nine varieties in the national marine fish industry technology system, Chinese tongue sole has made important contributions to China’s aquaculture industry (3). However, in recent years, due to the high density and intensive culture, vibriosis, caused by *Vibrio* species such as *V. harveyi*, has occurred frequently, resulting in high mortality rate (50%–70%), which has become one of the main bottlenecks restricting the healthy and sustainable development of Chinese tongue sole aquaculture industry (4). *V. harveyi* is also a prevalent bacterial pathogen in other mariculture species, such as crustaceans, shellfish, and fishes, resulting in high mortality rates and huge economic losses (5–7). Therefore, developing effective vibriosis targeted prevention and control measures, such as vaccines, probiotics, and selective breeding for disease resistance (8–11), is an urgent demand for the Chinese tongue sole industry’s advancement. To achieve this, we first need to identify specific intestinal microbes and host genes associated with vibriosis in Chinese tongue sole, and elucidate how they interact during the progression of vibriosis in Chinese tongue sole.

The intestinal microbiome plays important physiological roles in host immunity, nutrition, and disease susceptibility, and is also known as a “virtual organ” of the host, which is of great significance for maintaining host health (12, 13). This influence primarily depends on the structural stability and ecological balance of the intestinal microbial community. Intestinal dysbiosis occurs when opportunistic pathogenic bacteria exceed the threshold abundances, thereby leading to the onset of the disease (14). Significant alterations in the composition and abundance of intestinal microbiome during the development of bacteriosis or virosis were reported in a variety of aquatic animals, such as crustaceans (Chinese mitten crab (*Eriocheir sinensis*) (15), Pacific white shrimp (*Litopenaeus vannamei*) (16)) and fish (zebrafish (17), ayu (*Plecoglossus altivelis*) (18), *C. semilaevis* (19), Atlantic salmon (*Salmo salar*) (20), pearl gentian grouper (*Epinephelus lanceolatus* ♂ × *E. fuscoguttatus* ♀) (21)), which were detected by 16S rRNA sequencing technology. Yang et al. found that the intestinal microbiome of *Aeromonas hydrophila*-infected zebrafish has significantly changed: the abundance of some pathogenic bacteria genera including *Halomonas*, *Pelagibacterium*, and *Aeromonas* increased, while the beneficial bacteria taxa such as *Nitratireductor*, *Enterococcus*, and *Brevundimonas* decreased (17). Similarly, Huang et al. observed significant increases in *Proteobacteria*, *Firmicutes*, *Fusobacteria* in the intestinal microbiome of grass carp (*Ctenopharyngodon idellus*) challenged with *Aeromonas veronii* (22). Collectively, the above studies have validated that 16S rRNA sequencing is a powerful tool for characterizing the variations in the intestinal microbial community.

A comprehensive understanding of changes in host gene expression patterns during pathogen infection is essential for revealing the host’s immune molecular mechanism, and is critical for developing effective disease treatment strategies and enhancing resistance to pathogen infection (23). The gene expression profile of the host’s response to pathogen infection have been widely illustrated by RNA-seq technology. For example, Gao et al. identified novel gene expression patterns in mucosal immunity through transcriptomic analysis of the intestinal mucosa in *V. anguillarum* challenged turbot (*Scophthalmus maximus*) (23). Wang et al. demonstrated that DEGs between resistant and susceptible families of Japanese flounder (*Paralichthys olivaceus*) after infection with *Edwardsiella tarda* were predominantly enriched in immune-related pathways (24). Differential transcriptional responses between susceptible and resistant common carp (*Cyprinus carpio*) post infected with Cyprinid herpes virus-3 (CyHV-3) were found, which contributed to screen out the possible genes and pathways that may be involved in host disease resistance mechanisms (25). Taken together, RNA-seq not only advances our understanding of the molecular mechanisms of host immune defense, but also lays a theoretical foundation for the identification of candidate genes for disease-resistant selective breeding.

Alterations in intestinal microbiome composition and host gene expression during disease states are readily detectable; however, establishing causal relationships between these changes and disease pathogenesis remains challenging (26). Consequently, there is an urgent need to explore the interactions between intestinal microbiome and host gene expression during the development of disease. Nowadays, advances in high-throughput sequencing technology has made it possible to integrate microbiome and transcriptome analysis to unravel the interplay between intestinal microbiome and host gene, especially in human studies (27–29). For instance, Dayama et al. demonstrated that the expression of colorectal cancer-related genes (*LCN2* and *DUOX2*) is closely related to the abundance of colorectal cancer related bacteria (*Ruminococcaceae* and *Veillonella*) through the combined analysis of RNA-seq and 16S rRNA, indicating the potential role of host gene-microbe interactions in the pathogenesis of colorectal cancer in cystic fibrosis (27). In contrast, such integrated approaches are still rarely used in fish research. Zhou et al. revealed that the interactions between host gene and intestinal microbiome mediated through a microbe-intestine-immunity axis contribute to vibriosis resistance in Chinese tongue sole, employing the integrative analysis of metagenomic and transcriptomic (3).

A large number of studies have shown that host genetics has a significant effect on the intestinal microbiome composition (30–32). Therefore, to eliminate the variations in genetic background, we selected individuals from a full-sib family of Chinese tongue sole with the identical breeding conditions as the experimental model in the present study. Integrated histopathology, RNA-seq and 16S rRNA analyses were conducted on the posterior intestine from both resistant and susceptible individuals of Chinese tongue sole after being infected with *V. harveyi*. First, histopathological changes in the posterior intestinal tissues were compared between resistant and susceptible individuals. Subsequently, we explored the differences of intestinal microbiome and host gene expression in resistant and susceptible individuals, and identified specific microbes and host genes associated with vibriosis resistance and susceptibility. Finally, the interactions between the intestinal microbiome and host gene expression during *V. harveyi* infection were illustrated. The research results will provide new insights into the molecular mechanism by which the intestinal microbes interact with host genes to regulate the *Vibrio* resistance, and identify potential biomarkers for developing immunological or microbiome-based preventative or treatment therapies and selective breeding strategies against vibriosis in Chinese tongue sole.

## 2 Materials and methods

### 2.1 Fish and *V. harveyi* infection

To avoid the influence of environmental and host genetic factors on intestinal microbial composition and host gene expression, Chinese tongue sole individuals used in this study were selected from a full-sib family produced in 2022 and farmed in a same cement aquaculture pond at Flatfish Breeding Centre, Yantai, China. Three hundred heathy fish with a mean weight of 45.6 ± 2.3 g were randomly divided into ten tanks with 30 individuals per tank. After 7 days acclimation, 60 fish in two tanks were intraperitoneally injected with 0.1 ml 1× PBS, while the remaining 240 fish in other eight tanks were injected with 0.1 ml 2.5 × 10^5^ CFU/ml *V. harveyi* suspension.

### 2.2 Sample collection

Seven days after *V. harveyi* injection, 16 fish injected with PBS (set as control group), 16 fish with no vibriosis symptoms (set as resistant group), and 16 fish with slight or severe vibriosis symptoms (set as susceptible group) were selected for sampling. Posterior intestinal contents were aseptically collected from each sample, immediately frozen in liquid nitrogen, and stored at-80 °C for microbial DNA extraction and intestinal microbiome analysis. Concurrently, the posterior intestinal tissues from each fish were divided into two aliquots, one part fixed with 4% paraformaldehyde for histopathological analysis, and the other part was immediately frozen in liquid nitrogen and stored at-80 °C for transcriptomic analysis.

### 2.3 Histopathological analysis

All posterior intestinal tissue samples were fixed in 4% paraformaldehyde for more than 24 h. Then, the samples were dehydrated in alcohol, embedded in paraffin, and sliced into sections with a thickness of 4 μm using a Pathology slicer RM2016 (Leica, Wetzlar, Germany). Subsequently, the sections were dewaxed, stained with Hematoxylin and Eosin (HE), dehydrated through graded alcohols, and sealed with neutral gum. Finally, histopathological examination was performed using an upright optical Nikon Eclipse E100 microscope (Nikon, Tokyo, Japan) with digital image capture.

### 2.4 16S rRNA sequencing

Total DNA was extracted from posterior intestinal content samples using a TIANamp soil DNA kit (Tiangen, Beijing, China). Qualified DNA served as template for amplifying the V3-V4 region of bacterial 16S rRNA gene with specific barcoded primer (5’-CCTAYGGGRBGCASCAG-3’, 5’-GGACTACNNGGGTATCTAAT-3’). PCR products were mixed in equidensity ratios and purified. Subsequently, sequencing libraries were constructed and indexes were added. Then, the quantified libraries were pooled and subjected to paired-end sequencing on Illumina NovaSeq 6000 platform (Illumina, San Diego, USA) according to the effective library concentration and data amount required. Raw sequencing data have been uploaded to the National Center for Biotechnology Information (NCBI) Sequence Read Archive (SRA) database with accession number PRJNA1270419.

### 2.5 16S rRNA sequencing data processing and analysis

Paired-end reads were first merged using FLASH (v1.2.11) (33). The spliced tags were filtrated and removed chimera using fastp (v0.23.1) (34) and vsearch (2.16.0) (35), respectively. Then, denoising was conducted via DADA2 (36) to obtain Amplicon Sequence Variants (ASVs). QIIME2 (v202202) (36) was used for species annotation for each ASV against the Database of Silva 138.1. Relative abundance (RA) values was normalized for subsequent analysis. The intestinal microbes driving the differences between different groups at each taxonomic level were screened using t-test. Alpha diversity indices were calculated using QIIME2, and Kruskal-Wallis rank sum test was used for the intergroup difference assessment. Beta diversity index was quantified using weighted unifrac distances, and group differences was examined using Kruskal-Wallis rank sum test. Furthermore, principal co-ordinates analysis (PCoA) and non-metric multi-dimensional scaling (NMDS), both based on the weighted unifrac distance matrix, were also employed for the analysis and visualization of beta diversity.

### 2.6 Transcriptome sequencing

Total RNA were extracted from posterior intestinal tissues of 3 individuals per group using the RNA Easy Fast Tissue/Cell Kit (Tiangen; Beijng, China) according to the manufacturer’s instructions. RNA integrity and concentration were assessed with an Agilent 2100 bioanalyzer (Agilent Technologies, CA, USA). Pair-ended sequencing libraries were prepared using the TruSeq Stranded mRNA Library Prep Kit (Illumina, San Diego, USA) following the manufacturer protocols. After quality control, libraries were sequenced on Illumina NovaSeq x Plus platform.

### 2.7 RNA-seq data processing and analysis

Raw sequencing data were processed and quality controlled using fastp software (v0.23.1). Then, cleaned paired-end reads were aligned to the reference genome of *C. semilaevis* with Hisat2 (v2.0.5) (37). Transcript was quantified using Fragments Per Kilobase of transcript sequence per Millions (FPKM) by featureCounts (v1.5.0-p3) (38). Subsequently, DESeq2 (v1.20.0) (39) software was used to identify DEGs with the criteria of *q* value ≤ 0.05 and |log_2_ (fold change)| ≥ 1. Gene Ontology (GO) and Kyoto Encyclopedia of Genes and Genomes databases (KEGG) enrichment analyses were conducted with clusterProfiler (v3.8.1) (40), and significantly enriched GO terms and KEGG pathways with *q* value < 0.05 were identified. Simple Modular Architecture Research Tool (SMART) (https://smart.embl.de) was used to identify and annotate the protein domains.

### 2.8 Integrated analysis of the associations between intestinal microbes and host genes

To perform an integrated analysis of the intestinal microbiome and host genes, Spearman correlation analysis was conducted between differential intestinal microbes (at genus level) with RA ≥ 0.1% and coverage of > 10% samples and host DEGs significantly enriched in immune-related GO terms and KEGG pathways. The associations between intestinal microbes and host genes were evaluated by calculating the Spearman correlation coefficients (r) and the corresponding *p* values using the corr.test() function in R (v4.0.5). The Spearman correlations between the microbes significantly correlated (*p* value < 0.05) with the host genes were also computed.

## 3 Results

### 3.1 Histopathology of the posterior intestine

**Figure 1:**
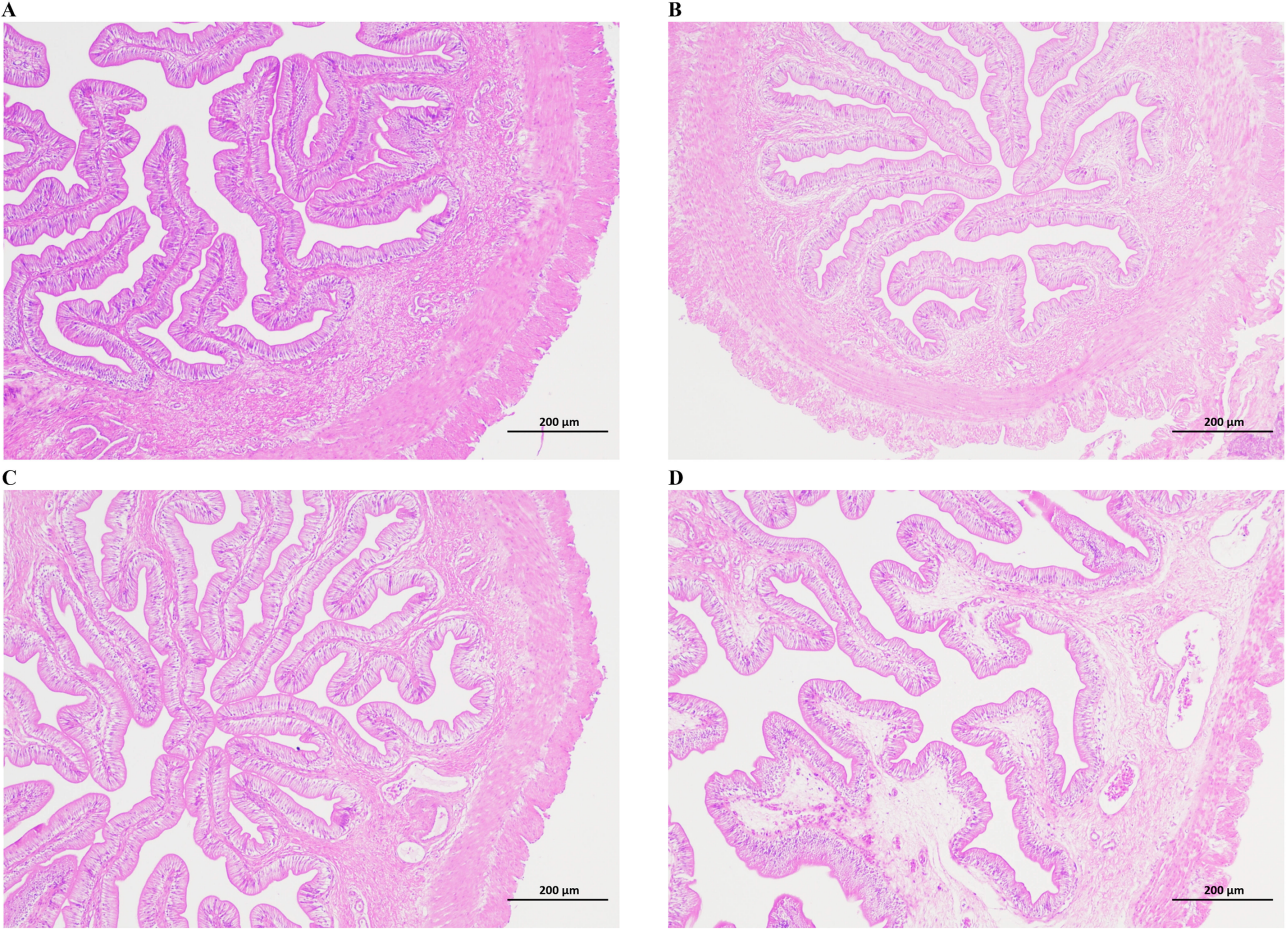
Tissue sections of the posterior intestine of *C semilaevis*. **(A)** Intestine tissues in the control group, **(B)** Intestine tissues in the resistant group, **(C, D)** Intestine tissues in the susceptible group.

As shown in **Figure 1A**, no histopathological changes were detected in the posterior intestinal tissues of Chinese tongue sole in the control group. In the resistant group, a small amount of inflammatory cell infiltration, a slight increase in both the number of goblet cells and the width of lamina propria were found in the intestinal tissues (**Figure 1B**). In contrast, obvious histopathological lesions were observed in the posterior intestinal tissues of the susceptible group. In detail, slight inflammatory cell infiltration, slight tissue dissociation of the mucosal layer and lamina propria, and a few vacuoles within the lamina propria were detected in slight susceptible individuals (**Figure 1C**). Meanwhile, in severely susceptible individuals, severe histopathological lesions were observed, including extensive inflammatory cell infiltration, severe tissue dissociation affecting the mucosal layer, submucosa, and lamina propria, and a significantly decreased thickness of the intestinal muscle layer accompanied by a great deal of necrotic areas (**Figure 1D**).

### 3.2 Differences in intestinal microbial community composition

**Figure 2:**
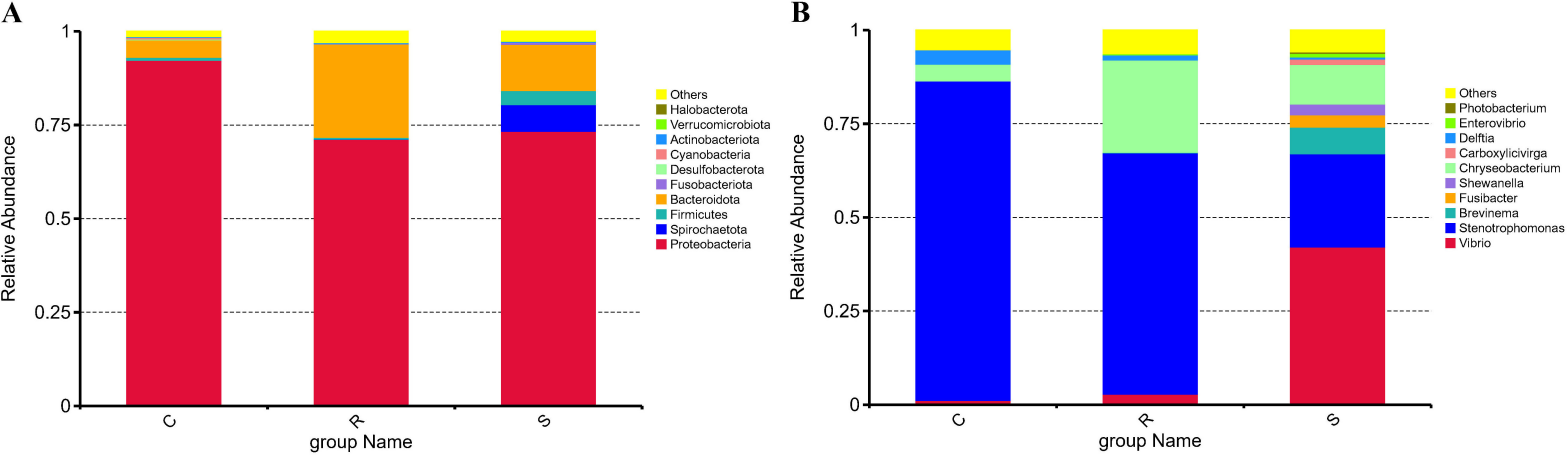
Relative abundances of the top 10 dominant bacterial phyla **(A)** and genus **(B)** in each group. C, R, and S represent control group, resistant group, and susceptible group, respectively (the same the following).

**Figure 3:**
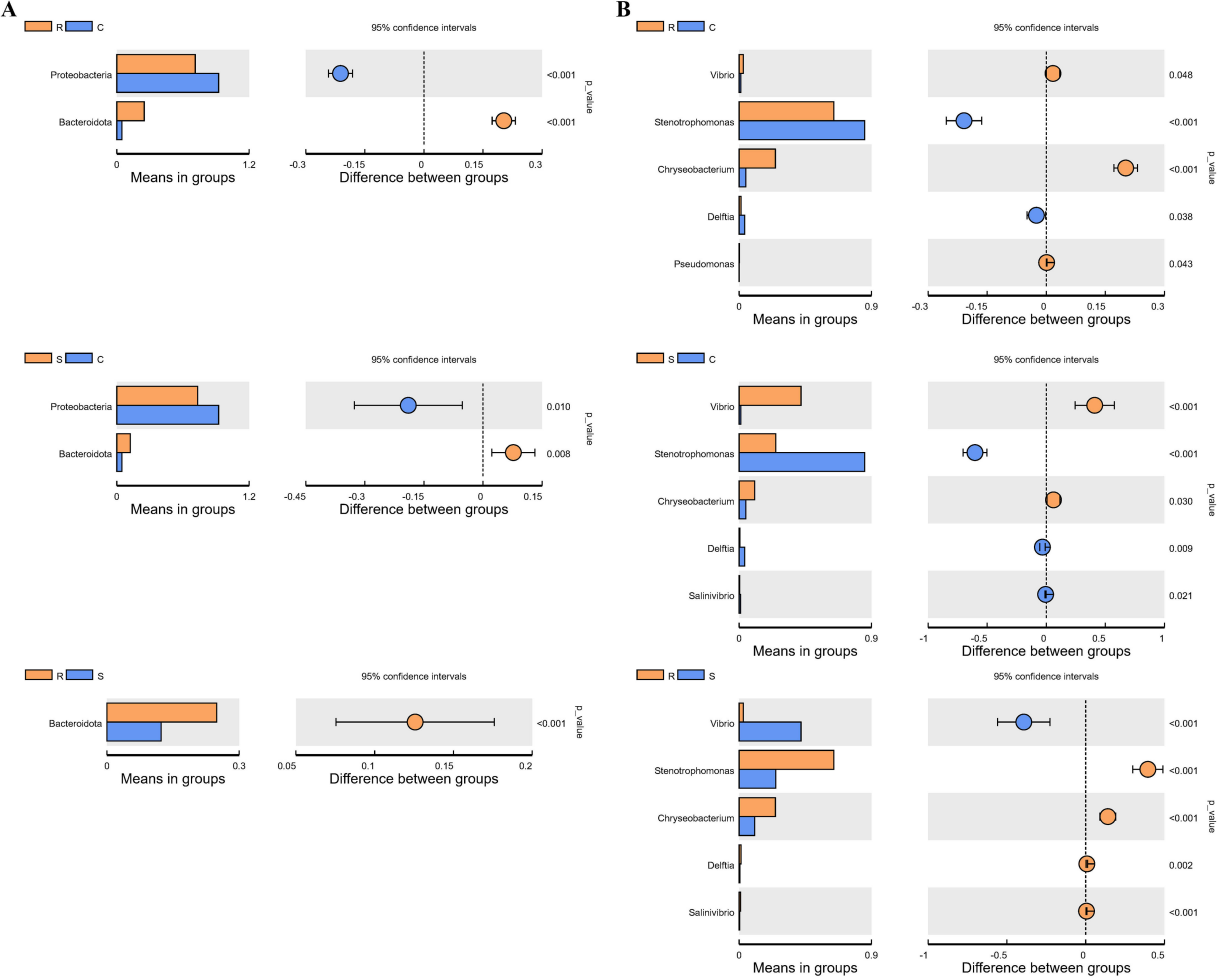
The microbial taxa with significant differences between groups at the phyla **(A)** and genus **(B)** level. The significance of the differences was tested with t-test.

After data quality control, a total of 5,163,419 effective reads were obtained from 48 samples, with an average of 107,571 ± 4,980 reads per sample (mean ± standard deviation) (**Supplementary Table S1**), resulting in 3182 unique ASVs (**Supplementary Table S2**) across all samples. These ASVs were aggregated to 35 phyla, 76 classes, 167 orders, 267 families, 511 genera, and 197 species. We selected the relative abundance at the phylum and genus level for further analysis. The relative abundances of the 10 most dominant bacterial phyla and the 10 most dominant genera were shown in **Figure 2**. Among these taxa, those with significant differences between groups were identified by t-test (**Supplementary Table S3**). At the phylum level, Proteobacteria was the most abundant across all groups, with its highest abundance in the control group (92.37%), significantly exceeding that in the resistant group (71.18%) and susceptible group (73.39%) (*p* < 0.01, t-test) (**Figure 3A**). In contrast, the abundance of Bacteroidota was significantly higher in the resistant group (24.89%) than that in the control (4.61%) and susceptible groups (12.32%) (*p* < 0.01) (**Figure 3A**). At the genus level, the abundance of *Stenotrophomonas* significantly higher in both the resistant (64.45%) and control groups (85.34%) compared to the susceptible group (24.96%) (*p* < 0.01) (**Figure 3B**). By comparison, *Vibrio* reached its highest abundance in the susceptible group (42.12%), which was significantly higher than that in the resistant (2.85%) and control groups (1.15%) (*p* < 0.01) (**Figure 3B**). In addition, the abundance of *Chryseobacterium* was highest in the resistant group (24.67%), and significantly higher than that in the control (4.51%) and susceptible groups (10.60%) (*p* < 0.01). Moreover, *Delftia* was the most abundant in the control group (3.81%), with significantly higher levels than that in the resistant (1.30%) (*p* < 0.05) and susceptible groups (0.54%) (t-test, *p* < 0.01) (**Figure 3B**). The abundance of *Salinivibrio* was comparable between the control group (1.00%) and resistant group (0.98%), but both were significantly higher than that in the susceptible group (0.44%) (t-test, *p* < 0.01) (**Figure 3B**). Other significantly differential genera between groups are listed in **Supplementary Table S3**.

**Figure 4:**
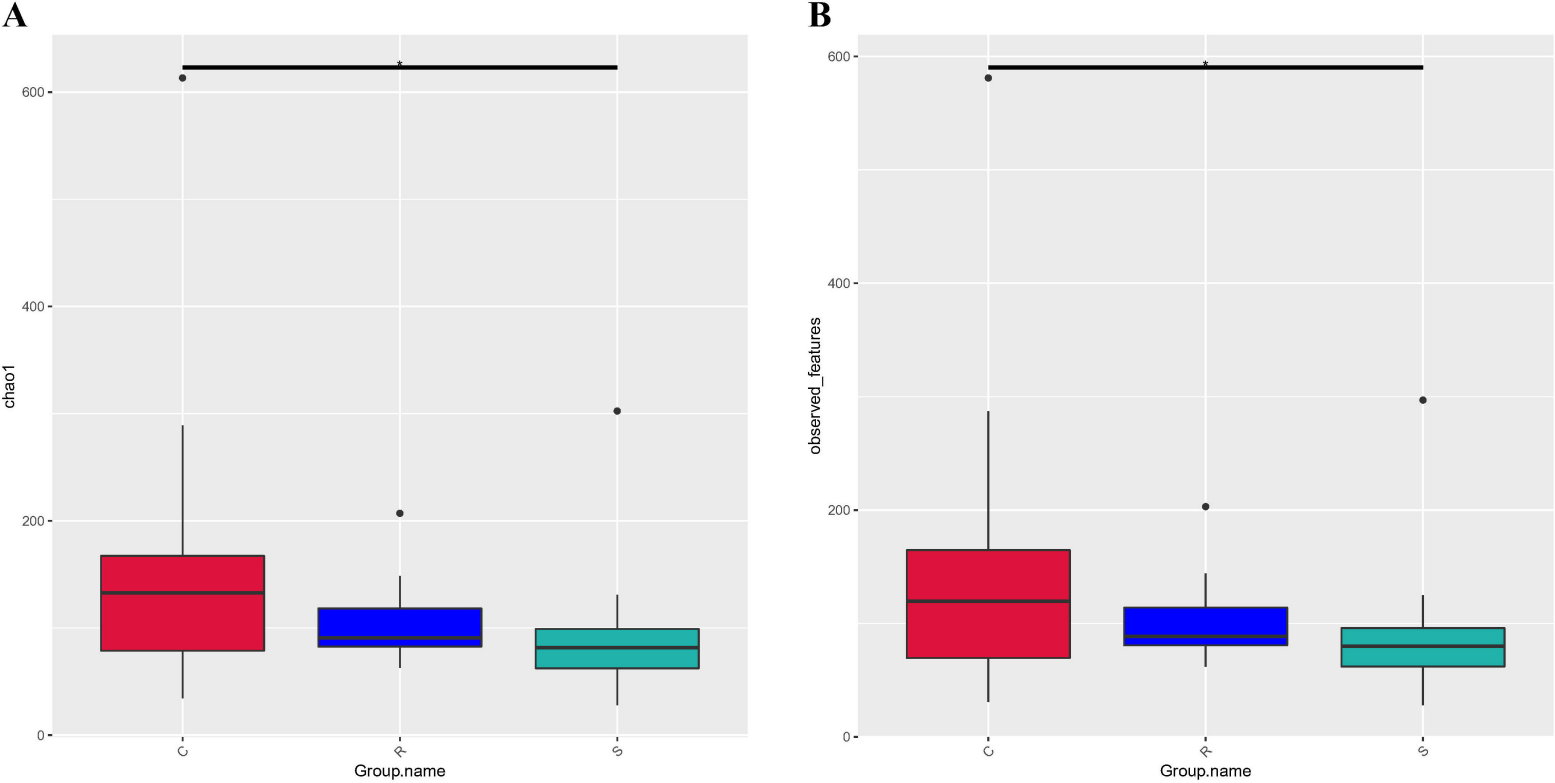
Boxplot depicting the difference of alpha diversity of intestine microbiome in the control, resistant and susceptible individuals of *C semilaevis*. **(A)** Chao 1 index. **(B)** observed features index. Kruskal-Wallis rank sum test was used to detect the variation between all groups.

**Figure 5:**
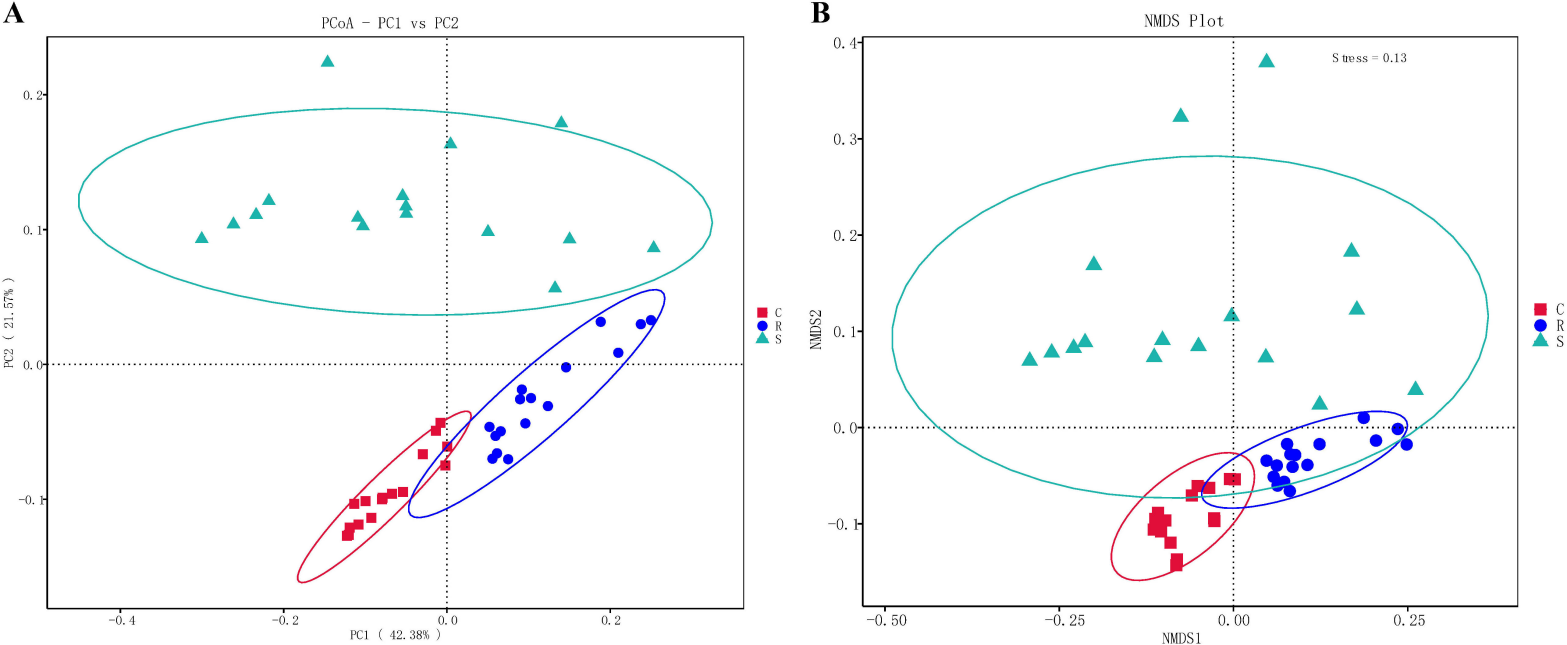
Beta diversity analysis of the microbial community structure between the control, resistant and susceptible groups. **(A)** PCoA plot based on Weighted_UniFrac distance matrix. **(B)** NMDS plot based on Weighted_UniFrac distance matrix. A stress of less than 0.2 indicates that NMDS can accurately reflect the degree of difference between samples.

Differences in intestinal microbial community between control, resistant and susceptible groups can also be assessed using alpha and beta diversity. Alpha diversity index (Chao1 and observed features) showed that the overall intestinal microbiome diversity decreased in the order of control group > resistant group > susceptible group (**Figure 4**). Specifically, both of these indexes were significantly lower in the susceptible group than in the control group (*p* < 0.05), but showed no statistically significant differences between control and resistant groups, and between resistant and susceptible groups (*p* > 0.05) (**Figure 4**). The difference analysis of beta diversity index between groups showed that there were significant differences between the susceptible group and the control group, and between the susceptible group and the resistant group, but no significant differences were detected between the control and resistant groups (**Supplementary Table S4**). Meanwhile, the differences among groups can also be visually confirmed in the PCoA and NMDS plots (**Figure 5**), where the samples were clearly clustered according to the disease status of control, resistant and susceptible individuals.

### 3.3 Host RNA-seq and DEGs identification

In order to reveal the distinct host response mechanisms in response to *V. harveyi* infection in resistant and susceptible individuals, comparative transcriptomic analysis was performed on the posterior intestine of Chinese tongue sole. A total of 56.06 Gb clean data were obtained from 9 samples, with an average Q30 value of 94.14% (**Supplementary Table S5**). The average reads mapping rate was 88.48% (**Supplementary Table S5**). The principal component analysis (PCA) plot showed clear clustering of the samples from the control, resistant and susceptible groups (**Supplementary Figure S1**), indicating differential gene expression patterns between resistant and susceptible individuals of Chinese tongue sole in response to *V. harveyi* infection. Raw RNA-seq data generated in this study were deposited in the NCBI SRA database with accession number PRJNA1270417.

**Figure 6:**
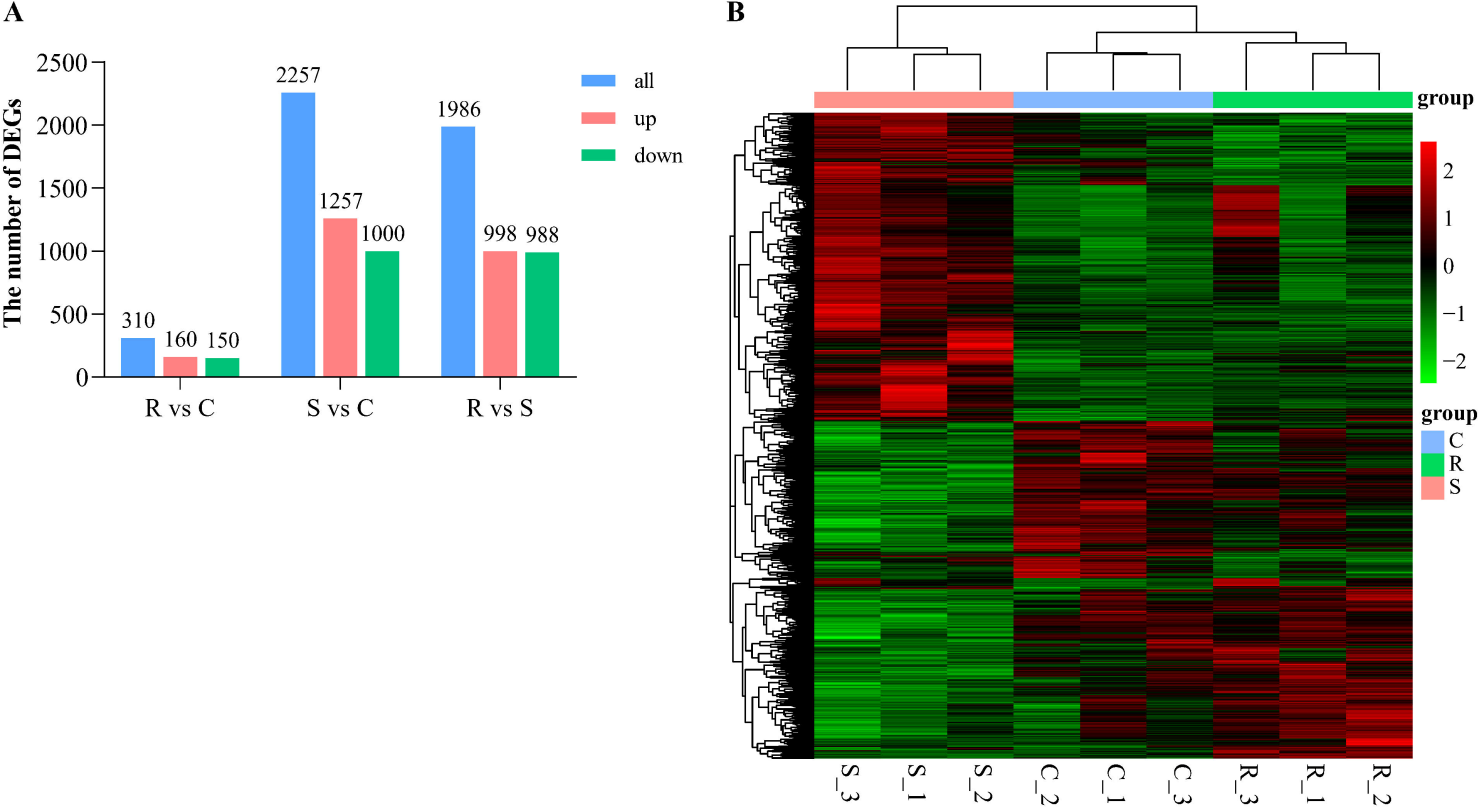
The DEGs analysis in posterior intestine of *C semilaevis* post infection with *V. harveyi*. **(A)** Statistics of DEGs in different compare groups, including R vs. C, S vs. C and R vs. S. Up and down-regulated genes were colored in red and blue, respectively. **(B)** The heat map for DEGs clustering in all groups. The x axis is the sample name, and the y axis is the normalization FPKM value of DEGs.

By comparing the gene expression profiles of the host posterior intestine tissues, 310, 2,257, and 1,986 host DEGs were detected in the resistant group vs. the control group, the susceptible group vs. the control group, and the resistant group vs. the susceptible group, respectively (**Figure 6A**). Compared to the control group, 160 and 1,257 genes were upregulated in the resistance group and the susceptible group, while 150 and 1,000 genes were downregulated in the resistance group and the susceptible group, respectively. In addition, among the 1,986 DEGs in the resistant group vs. the susceptible group, 998 were upregulated and 988 were downregulated. The hierarchical cluster analysis of all DEGs showed high intergroup difference and obvious intragroup similarity in expression patterns (**Figure 6B**). The full list of DEGs can be available in **Supplementary Table S6**.

### 3.4 Function analysis of host DEGs

**Figure 7:**
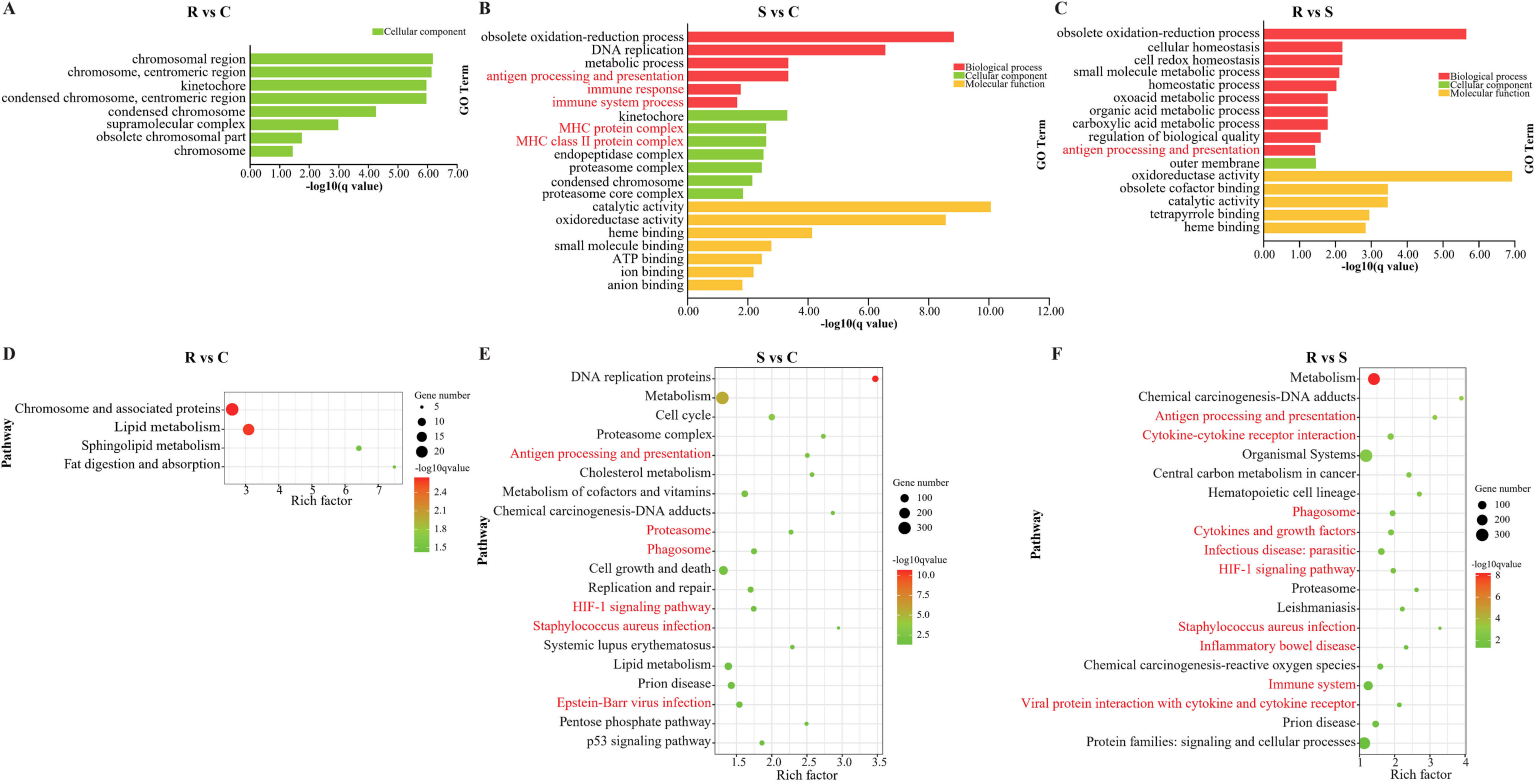
The significant enriched GO terms and KEGG pathways of DEGs in *C semilaevis* post infection with *V. harveyi*. **(A, D)** DEGs in R vs. C, **(B, E)** DEGs in S vs. C, **(C, F)** DEGs in R vs. S. Red font represent immune-related pathways.

To further investigate the differences in gene function and immune regulatory mechanisms between resistant and susceptible individuals after infection with *V. harveyi*, GO and KEGG enrichment analyses were performed on host DEGs, focusing on immune-related GO terms and pathways. The GO enrichment analysis results were list in **Supplementary Table S7**. The results showed that host DEGs in resistant group vs. control group were significantly enriched in cellular component GO terms, including chromosomal region, kinetochore, and supramolecular complex (*q* < 0.05) (**Figure 7A**). Host DEGs in susceptible group vs. control group showed significant enrichment in immune-related GO terms, such as antigen processing and presentation, MHC protein complex, MHC class II protein complex, immune response, and immune system process (*q* < 0.05) (**Figure 7B**). Significantly enriched GO terms of host DEGs in resistant group vs. susceptible group included cellular homeostasis, homeostatic process, and antigen processing and presentation (*q* < 0.05) (**Figure 7C**). Simultaneously, some other immune-related GO terms, including immune response, immune system process, were also enriched (*p* < 0.05) (**Supplementary Table S7**).

The detailed information of KEGG enrichment analysis can be obtained in **Supplementary Table S8**. The result showed that host DEGs in resistant group vs. control group were significantly enriched in pathways, such as lipid metabolism, chromosome and associated proteins, and fat digestion and absorption (*q <*0.05) (**Figure 7D**). Host DEGs in susceptible group vs. control group showed significant enrichment in multiple immune-related pathways, including antigen processing and presentation, phagosome, cell growth and death, proteasome, Staphylococcus aureus infection, and Epstein-Barr virus infection (*q <*0.05) (**Figure 7E**). Meanwhile, some other immune-related pathways, such as complement and coagulation cascades, inflammatory bowel disease and immune disease, were also enriched (*p* < 0.05) (**Supplementary Table S8**). In contrast, host DEGs between the resistant group and the susceptible group were significantly enriched in more immune-related pathways, such as antigen processing and presentation, cytokine-cytokine receptor interaction, phagosome, cytokines and growth factors, inflammatory bowel disease, immune system, and viral protein interaction with cytokine and cytokine receptor (*q <*0.05) (**Figure 7F**).

**Figure 8:**
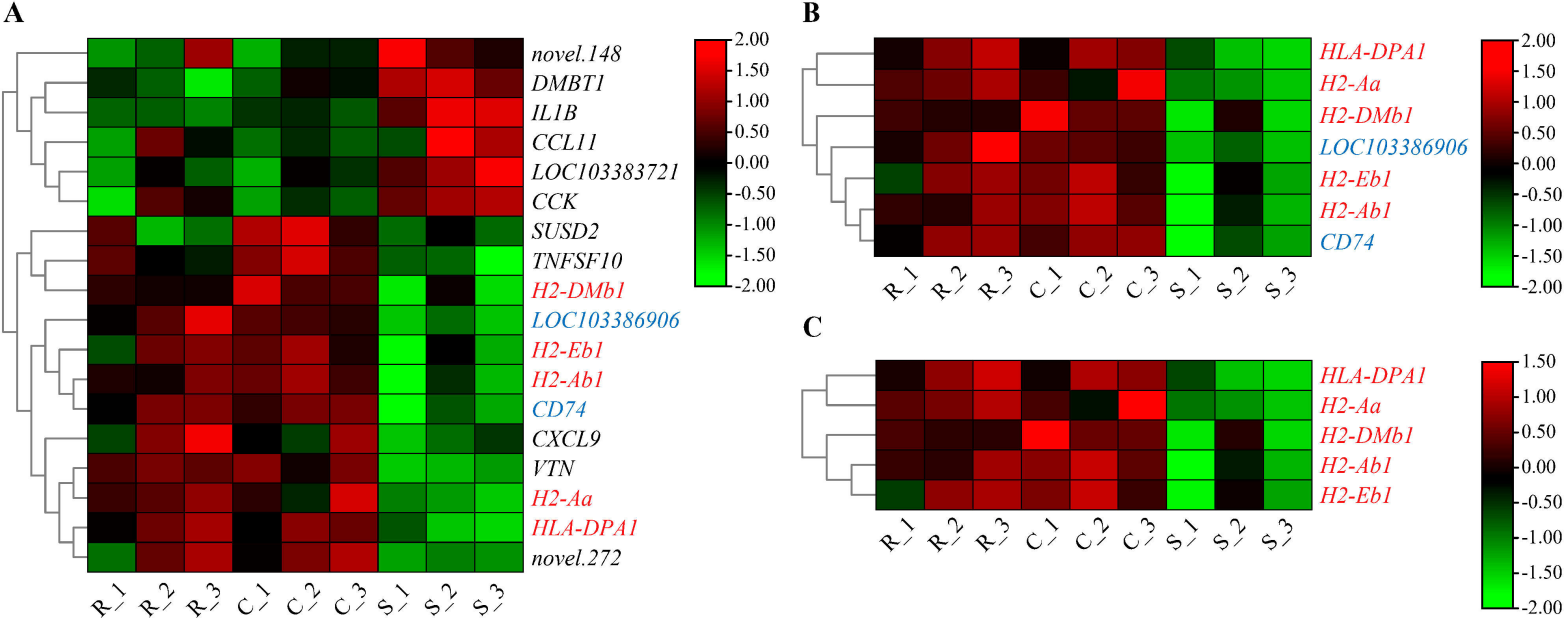
Expression patterns of DEGs in immune-related GO terms of **(A)** immune system process and immune response, **(B)** antigen processing and presentation, **(C)** MHC protein complex and MHC class II protein complex. The red and blue fonts represent DEGs that coexist in at least five and three GO terms or pathways, respectively. Cells with different colors correspond to different expression levels, which were normalized into log_2_(FPKM + 1) (the same the following).

**Figure 9:**
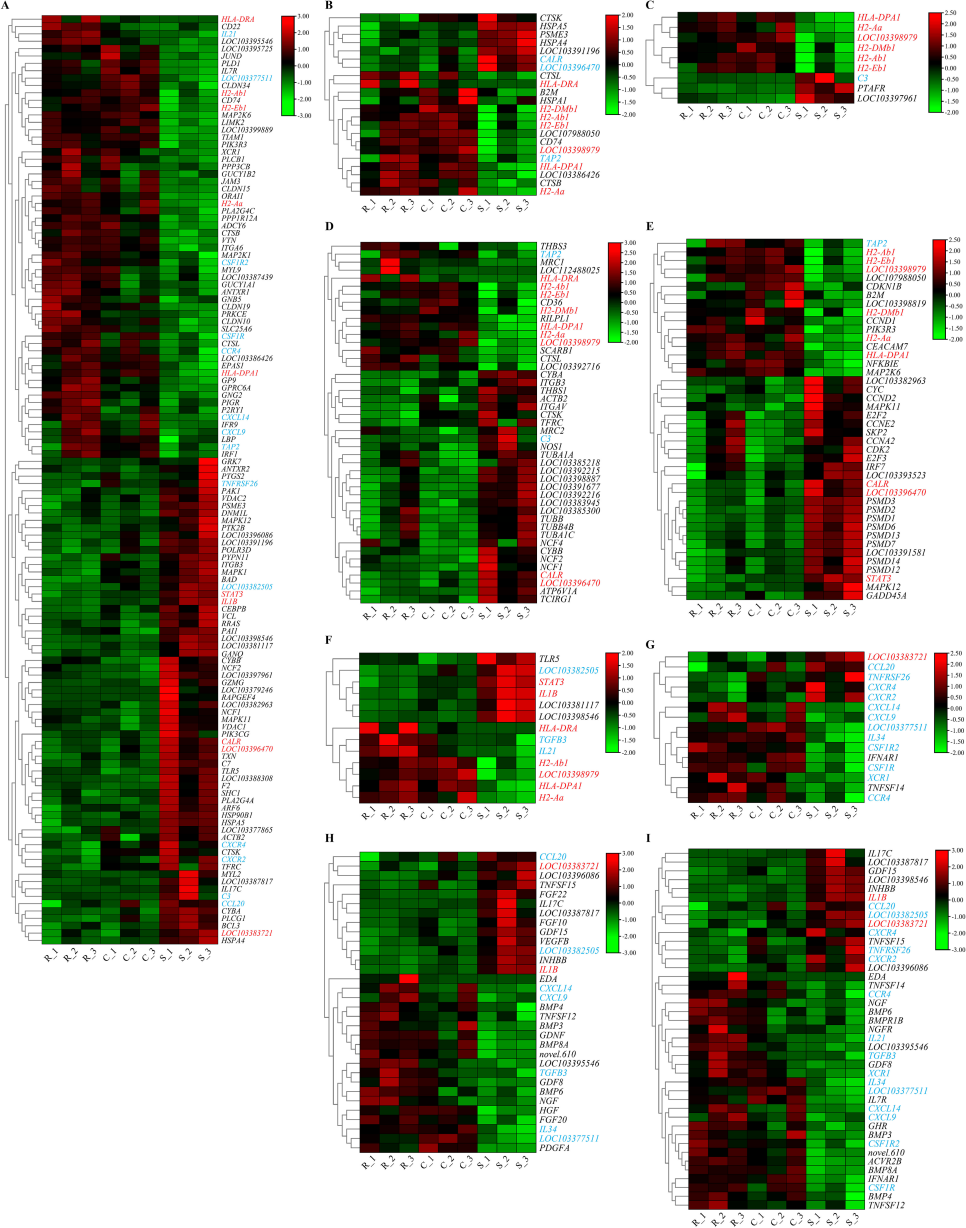
Expression patterns of DEGs in some of immune-related pathways of **(A)** immune system, **(B)** antigen processing and presentation, **(C)** Epstein-Barr virus infection, **(D)** phagosome, **(E)** Staphylococcus aureus infection, **(F)** inflammatory bowel disease, **(G)** viral protein interaction with cytokine and cytokine receptor, **(H)** cytokines and growth factors, and **(I)** cytokine-cytokine receptor interaction. The red and blue fonts represent DEGs that coexist in at least five and three pathways, respectively.

**Figure 10:**
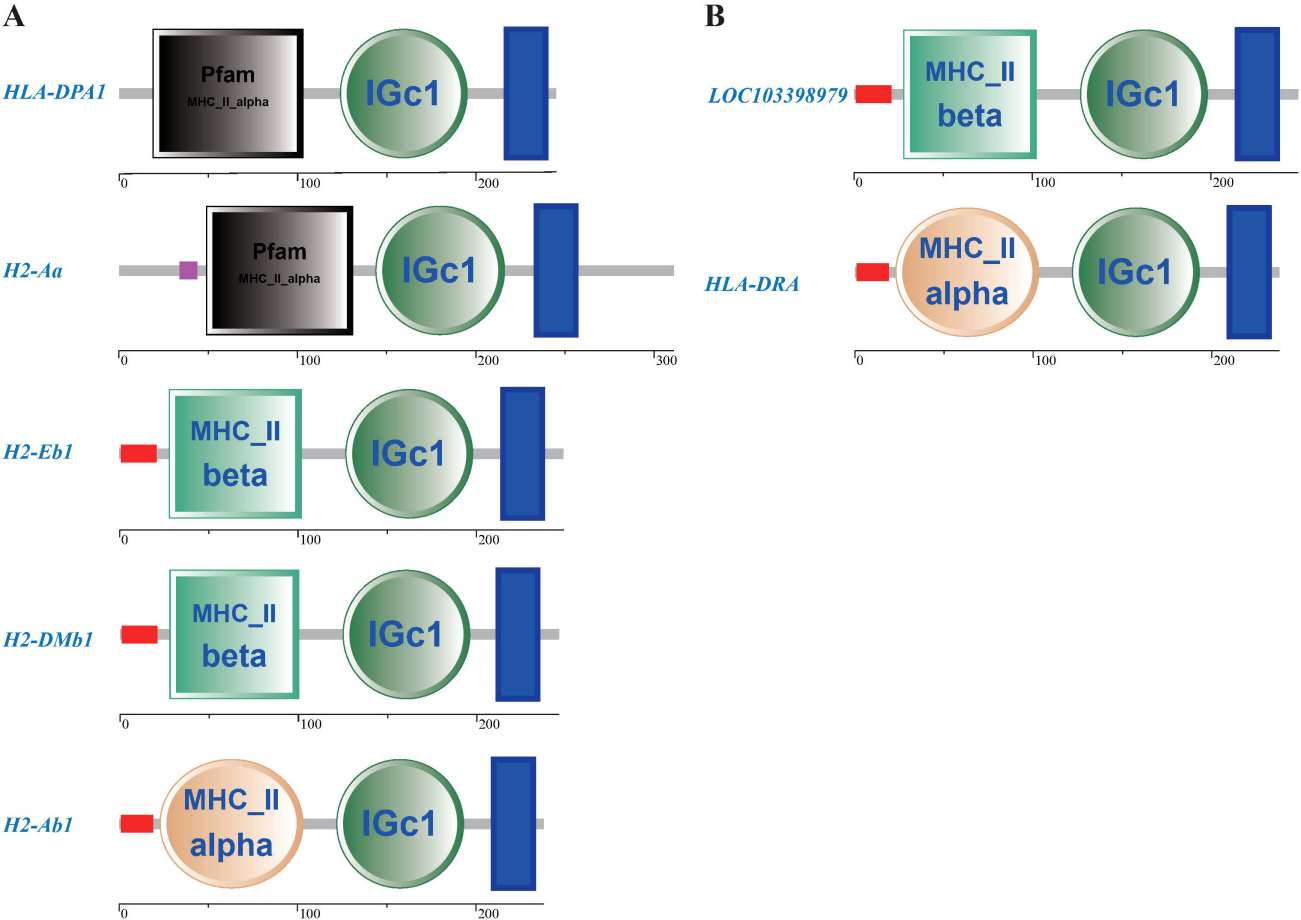
The structural characteristics of DEGs that coexist in immune-related GO terms and pathways and are upregulated in the control and resistant groups compared to the susceptible group. **(A)** DEGs coexisting in at least five immune-related GO terms and five immune-related pathways. **(B)** DEGs coexisting in at least five immune-related pathways.

According to the results of GO and KEGG enrichment analysis, a total of 285 host DEGs significantly enriched in immune-related GO terms and pathways were screened, and they showed markedly different expression patterns between the resistant and susceptible groups (**Figure 8** and **Figure 9**). It is worth noting that many of these DEGs coexisted in different immune-related GO terms and pathways. For example, *HLA-DPA1*, *H2-DMb1*, *H2-Aa*, *H2-Ab1*, and *H2-Eb1* simultaneously existed in at least five immune-related GO terms and five immune-related pathways, and *LOC103398979* and *HLA-DRA* coexisted in at least five immune-related pathways. Notably, these seven genes were all upregulated in the resistant and control groups when compared to the susceptible group, and they all belonged to the MHC class II gene family and possessed a same immunoglobulin C-Type (IGc1) domain and a same transmembrane region (**Figure 10**). Furthermore, *IL1B*, *STAT3*, *CALR*, *LOC103396470*, and *LOC103383721* were present in at least five immune-related pathways, and these genes were upregulated in the susceptible group. In addition, *CCR4*, *TAP2*, *CXCL9*, *CXCL14*, *TGFB3*, *LOC103377511*, *CSF1R*, *CSF1R2*, *IL21*, *IL34*, and *XCR1* existed in at least three immune-related pathways, and they were upregulated in the resistant and control groups. Moreover, *CCL20*, *C3*, *LOC103382505*, *TNFRSF26*, *CXCR4*, and *CXCR2* existed in at least three immune-related pathways, but they were downregulated in resistant group. Besides, *CD74* and *LOC103386906* were existed in at least three immune-related GO terms, and they were upregulated in the resistant group. In conclusion, these coexisting host genes exhibited divergent expression patterns in resistant and susceptible individuals of after infection with *V. harveyi*, indicating their pivotal roles in mediating the resistance divergence to vibriosis in Chinese tongue sole.

### 3.5 Correlations between differential intestinal microbes and host DEGs

**Figure 11:**
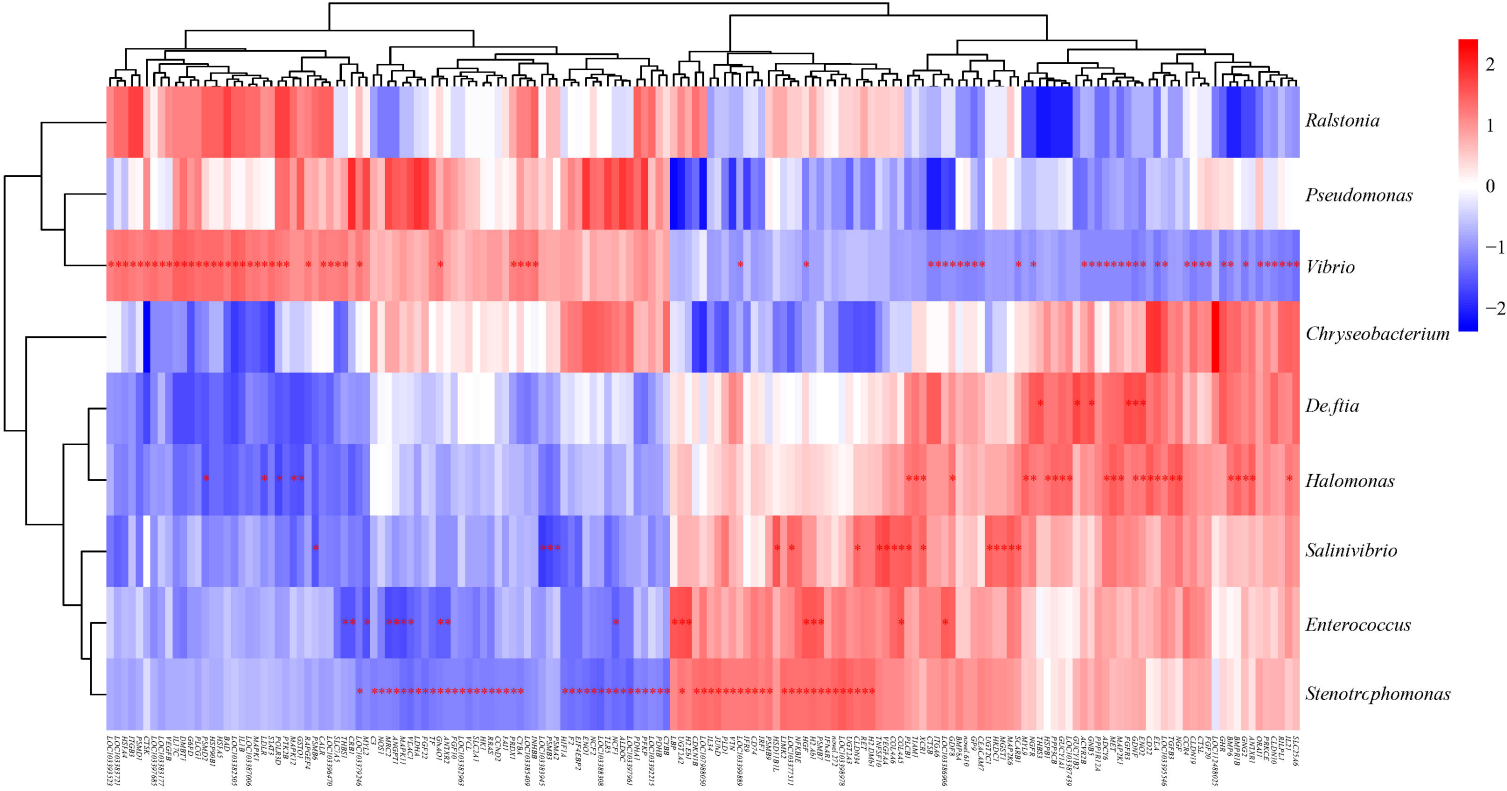
Interactions between host DEGs and intestinal microbes. DEGs enriched in immune-related GO terms and KEGG pathways, and differential intestinal microbes (at genus level) with RA ≥ 0.1% and coverage of > 10% samples were selected for correlation analysis. The color of the cells indicates the magnitude of the correlation. * indicate significant Spearman correlation with *p* value < 0.05 and |r| ≥ 0.75.

To investigate the potential roles of host gene-intestinal microbiome interactions in the pathogenesis of vibriosis, the correlations between 9 differential intestinal microbes (*p* < 0.05, at the genus level) and 284 host DEGs enriched in immune-related GO terms and KEGG pathways and were explored (**Supplementary Table S9**). As a result, 207 strong gene-microbe correlations were detected (*p* value < 0.05), where the |r| ≥ 0.75 (labeled with “*” in **Figure 11**). Among these, we found some significant positive gene-microbe correlations, between *H2-Ab1* and *Stenotrophomonas* (r = 0.85), *H2-DMb1* and *Stenotrophomonas* (r = 0.85), *IL17C* and *Vibrio* (r = 0.97), *IL1B* and *Vibrio* (r = 0.93), *TGFB3* and *Halomonas* (r = 0.91), *IL34* and *Stenotrophomonas* (r = 0.82), *CD74* and *Stenotrophomonas* (r = 0.80). Meanwhile, some negative correlations were also detected, such as between *EDA* and *Vibrio* (r =-0.86), *ITGA6* and *Vibrio* (r =-0.80), *TLR5* and *Stenotrophomonas* (r =-0.87), *C3* and *Stenotrophomonas* (r =-0.80), IL21 and *Vibrio* (r =-0.83), and *MRC2* and Enterococcus (r =-0.85). In addition, strong microbe-microbe correlations (|r| ≥ 0.6, *p* value < 0.05) among microbes that were significantly correlated with host genes were also found, including the negative correlations between *Vibrio* and *Stenotrophomonas* (r =-0.76), and *Vibrio* and *Delftia* (r =-0.61).

## 4 Discussion

Chinese tongue sole is an economically important flatfish species widely cultivated in China. However, the outbreak of vibriosis in recent years, especially caused by *V. harveyi*, has led to a high mortality and enormous economic losses, seriously hindering the development of the Chinese tongue sole farming industry. Therefore, this study aims to explore the changes in intestinal histopathology, intestinal microbial composition, and host gene expression between resistant and susceptible individuals, as well as the interactions between intestinal microbes and host genes during the progression of vibriosis, thereby providing new insights for the prevention and treatment of vibriosis in Chinese tongue sole. It is worth mentioning that in order to eliminate the influence of host genetics on the intestinal microbiome (32, 41), individuals from a full-sib family were selected as the research subjects.

### 4.1 Pathological changes of the posterior intestine in Chinese tongue sole after *V. harveyi* infection

The intestine is the first barrier in fish to prevent the invasion of pathogens (42). In recent years, some studies have shown that pathogen infection tends to cause pathological damage to the intestine of fish. For instance, after infection with *V. vulnificus*, the intestine of grass carp (*Ctenopharyngodon idellus*) exhibits distinct pathological changes, including thinning of the membrana serosa, infiltration of inflammatory cells into the submucosa, and the distribution of basophilic granulocytes in the lamina propria (43). Obvious pathological lesions were detected in the intestine of Chinese tongue sole after challenging with *V. anguillarum*, such as the tissue separation of mucosal layer and lamina propria, the tissue dissociation of the submucosa, and the infiltration of inflammatory cells (44). However, studies on the pathological damage of the intestine in Chinese tongue sole after *V. harveyi* infection have not been reported so far. In this study, histopathological observation revealed that the posterior intestines of resistant and susceptible individuals exhibited obviously distinct pathological damages post infection with *V. harveyi*. Pathological lesions in the susceptible group were more severe than those in the control and resistant groups, such as a significant increase in the inflammatory cell infiltration, increased tissue dissociation of mucosal layer and lamina propria, and the occurrence of necrotic areas. In addition, the degree of damage in the posterior intestines is consistent with the degree of vibriosis symptoms observed from the appearance. The above results suggest that the invasion of *V. harveyi* promotes the occurrence of vibriosis, and there may be significant differences in the intestinal barrier function between the resistant and susceptible individuals.

### 4.2 Variations in intestinal microbial community composition in Chinese tongue sole induced by *V. harveyi* infection

A large number of studies have shown that intestinal microbial diversity is positively correlated with host health condition, and the diversity of intestinal microbes significantly decreases during the progression of host diseases (18, 21, 45). Similar results were found in our study, namely that the intestinal microbial alpha diversity indexes (Chao1 and observed features) in the susceptible group were lower than those in the control and resistant groups. Thus, increasing the diversity of the microbiome may potentially contribute to maintaining the host’s health. Beta diversity analysis indicated that there were significant differences in the intestinal microbial composition between the susceptible group and the control group, as well as between the susceptible group and the resistant group. Meanwhile, the PCoA and NMDS analyses also provided evidence for these differences, where the samples were clearly clustered according to the symptoms of vibriosis. Similarly, PCoA analysis for the intestinal microbes of pearl gentian grouper (*Epinephelus lanceolatus*♂× *E. fuscoguttatus* ♀) infected with *V. harveyi* could separate the high-virulence *V. harveyi* group from the low-virulence and control groups (21). These findings imply that *V. harveyi* infection accelerates the dysbiosis of the intestinal microorganisms and drives the differences in the intestinal microbial community composition between the resistant and susceptible individuals of Chinese tongue sole, accompanied by changes in the microbial diversity.

In this study, the microbial taxa with differences between groups were identified at the phylum and genus levels. At the phylum level, the composition of intestinal microbial community in each group was dominated by Proteobacteria, which was consistent with the findings in other teleosts, such as Chinese tongue sole (19), zebrafish (46), and freshwater fish species (*Ctenopharyngodon idellus*, *Siniperca chuatsi* and *Silurus meridionalis*) (47). Moreover, Bacteroidota was detected with significantly high abundance in the resistant group. Although some members of Bacteroidota may be opportunistic pathogens, many Bacteroidota bacteria are symbiotic species highly adapted to the gastrointestinal tract. For example, Bacteroidota species are selectively recognized by the immune system of the host through specific interactions (48). Of course, the role of Bacteroidota in fish’s resistance to vibriosis requires further research and verification. At the genus level, after infection with *V. harveyi*, the abundances of *Vibrio* and *Pseudomonas* significantly increased, whereas the abundances of *Stenotrophomonas*, *Chryseobacterium*, *Delftia*, *Salinivibrio*, and *Enterococcus* significantly decreased. *Vibrio* is a well-known opportunistic pathogen associated with vibriosis in a variety of marine animals, such as fish (49), shellfish (50), and crustaceans (5). It is reported that the overabundance of *Vibrio* in the intestine of various teleosts, including ayu (18), Chinese tongue sole (19), and pearl gentian grouper (21), occurs simultaneously with the progress of the disease. In this study, *Vibrio* showed the highest abundance in the susceptible group, which is associated with the occurrence of vibriosis in susceptible hosts and reflects the significant differences in resistance to *V. harveyi* infection between the resistant and susceptible individuals. *Stenotrophomonas* plays key roles in fields such as environmental remediation, enzyme production, agriculture and disease treatment (51, 52). Ding et al. found that *Stenotrophomonas acidaminiphila* can significantly alleviate colonic shortening caused by DSS-induced colitis, and also has a strong ability to resist oxidation, inflammation and improve the intestinal mucosal barrier (53). For *Chryseobacterium*, some members of this genus are pathogenic to fish, but they are not relevant pathogens for domestic animal species in veterinary medicine (54). In addition, *Chryseobacterium indologenes* SR50 was reported to have potent anti-herpes simplex virus-1 activity in plants (55). Many strains of *Delftia* have agricultural and industrial relevance, including plant-growth promotion, bioremediation of hydrocarbon-contaminated soils, and heavy metal immobilization (56). For instance, *Delftia tsuruhatensis*, strain HR4, isolated from the rhizoplane of rice could suppress the growth of various plant pathogens effectively, especially the three main rice pathogens (*Xanthomonas oryzae* pv. *oryzae*, *Rhizoctonia solani* and *Pyricularia oryzae Cavara*) (57). *Delftia acidovorans* selectively coaggregates with partner bacteria and plays an important role in providing a metabolic opportunity for partner bacteria (58). The beneficial effects of *Stenotrophomonas*, *Chryseobacterium* and *Delftia* on aquatic animals have not been reported so far. *Salinivibrio* species can be selected as a potential probiotics, because they can regulate the expression of *irs1* and *foxo1* by increasing the level of 5’- adenylic acid, thereby promoting the growth of fish (59). The potent anti-parasitic activity of *Salinivibrio proteolyticus* strain YCSC6 was revealed by integration of metabolomics and transcriptomics (60). In this study, *Stenotrophomonas*, *Chryseobacterium*, *Delftia* and *Salinivibrio* exhibited significantly higher prevalence in the resistant group than that in the susceptible group, indicating that these genera may have potential roles in *V. harveyi* resistance in resistant individuals, but their roles remain to be further validated. On this basis, safe probiotic strains with the potential to resist *V. harveyi* may be screened out.

### 4.3 Changes in gene expression of Chinese tongue sole after *V. harveyi* infection

Several previous transcriptome analyses have studied the immune response of Chinese tongue sole against *V. harveyi* infection, mainly focusing on specific immune-related tissues or comparisons between resistant and susceptible families (3, 61, 62). In the current study, we conducted the first comparative transcriptome analysis of gene expression differences between resistant and susceptible individuals within the same family at 7 days post infection with *V. harveyi*. More DEGs were detected in the susceptible group than in the resistant group compared to the control group. Distinct transcriptional responses to infections between susceptible and resistant individuals in other fish species have also been reported. For example, more DEGs were detected in susceptible common carp (*Cyprinus carpio*) than in resistant individuals at 4 days after Cyprinid herpes virus-3 (CyHV-3) infection (25). Similarly, the number of DEGs in susceptible Atlantic salmon individuals at 7 days after Infectious Pancreatic Necrosis virus (IPNV) infection is twice that of resistant individuals (63). We speculate that the resistant individuals underwent significant transcriptional changes in the early stage of infection and then returned to normal levels on the 7th day after *V. harveyi* infection, whereas the susceptible individuals exhibit escalating gene expression changes as vibriosis progresses. The results of some other studies also support this inference. For instance, a large number of DEGs were concentrated at 6 – 24 h in both spleen and liver of *Plectropomus leopardus* after *V. harveyi* infection (64). Furthermore, the largest number of DEGs was detected in Chinese tongue sole at 16 h after *V. harveyi* infection (62). These results demonstrate distinct transcriptional responses to *V. harveyi* infection in the resistant and susceptible individuals.

In order to further understand the differences in resistance mechanisms between resistant and susceptible individuals, further functional enrichment analyses at the levels of GO term and pathway were conducted on DEGs. In susceptible individuals, host DEGs predominantly enriched in immune-related GO terms, such as antigen processing and presentation, MHC protein complex, immune response, and immune system process, and immune-related pathways, such as antigen processing and presentation, phagosome, and Staphylococcus aureus infection. In contrast, DEGs in resistant individuals showed no significant enrichment in immune-related GO terms or pathways. We speculate that this disparity arises because the immune response of resistant individuals occurs at the early stage of infection. Therefore, when sampled on the 7th day after infection with *V. harveyi*, their transcriptional levels had returned to normal, which was consistent with the decrease in the number of DEGs in the resistant group at this sampling point. Similarly, significantly enriched innate immune signaling pathways were observed in both spleen and liver of *P. leopardus* during early infection (6 – 12 h) (64). In contrast, DEGs in the spleen were significantly enriched in the pathways related to cellular processes and DNA repair at the middle and late infection stages (64). Furthermore, immune-related GO terms, including defense responses and immune responses, were enriched only at 16 h post infection with *V. harveyi*, while immune-related pathways, including antigen processing and presentation and cytokine-cytokine receptor interaction, were detected at 16 h and 48 h in Chinese tongue sole after *V. harveyi* infection (62). However, for susceptible individuals, the immune response may run through the entire process of infection and may intensify as the severity of the disease progression. For instance, higher interferons response (categorized under the GO term ‘response to virus’) was observed in susceptible Atlantic salmon at 7 and 21 days post infection with IPNV (63) and at the late stage of infectious salmon anemia virus (ISAV)infection (65). These findings potentially suggest that the differences in resistance between resistant and susceptible individuals may be closely related to the timing of immune response activation, and the early activation of the immune response may enhance disease resistance. Thus, the transcriptional responses in resistant and susceptible individuals of Chinese tongue sole at the early stage of *V. harveyi* infection need to be further explored.

DEGs that are significantly enriched in immune-related GO terms and pathways, especially those coexisting in multiple immune-related GO terms and pathways, should be given special attention. In this study, *HLA-DPA1*, *H2-DMb1*, *H2-Aa*, *H2-Ab1*, *H2-Eb1*, *LOC103398979* and *HLA-DRA* coexisted in at least five immune-related GO terms or pathways, and they all belonged to the MHC class II gene family, sharing an identical IGc1 domain and transmembrane region. MHC molecules play important roles in the immunity of vertebrate, and they can achieve successful presentation of antigenic fragments to the immune system through various biochemical reactions (66, 67). These seven genes were all upregulated in the resistant group after *V. harveyi* infection, which was consistent with the findings in *S. maximus* and *P. leopardus*, confirming their importance in antibacterial defense (64, 68). Therefore, it is necessary to further validate their roles in resistance against *V. harveyi* and select them as candidate markers for molecular design breeding aimed at developing resistance to *V. harveyi* infection. Chemokines play a core role in the development and homeostasis of the immune system, participating in all innate and adaptive immune responses and inflammatory responses by coordinating the migration of immune cells from lymphoid organs towards the sites of infection or tissue damage (69, 70). In this study, the chemokines genes (*CXCL9*, *CXCL14*, and *CCL20*) and their receptors (*CCR4*, *CXCR2*, and *CXCR4*), co-occurring in at least three immune-related pathways, showed significantly distinct expression patterns between the resistant and susceptible groups. This indicates substantial differences in the immune cell activation and migration coordination between resistant and susceptible individuals of Chinese tongue sole. As pro-inflammatory cytokines, interleukins (ILs) can regulate numerous biological processes and immune response, including the activation and differentiation of immune cells and their proliferation, maturation, migration, and adhesion during inflammatory processes and immune responses (71, 72). In this study, *IL1B*, *IL21*, and *IL34*, which were present in at least three immune-related pathways, were significantly upregulated in the resistant group after *V. harveyi* infection, demonstrating that a stronger inflammatory immune response could be activated in resistant individuals than in susceptible individuals to resist the invasion of *V. harveyi*. Collectively, these results suggest that these genes that coexist in immune-related GO terms and pathways and show significantly different expression patterns in resistant and susceptible individuals may contribute to the formation of *V. harveyi* resistance differences in Chinese tongue sole.

### 4.4 Interactions between the intestinal microbiome and host gene in vibriosis

Previous studies have reported changes in the composition of intestinal microbial and host genes expression after *Vibrio* infection in Chinese tongue sole, but these results all relied on a single type of omics data (19, 61, 62, 73). So far, the integrated omics analysis of intestinal microbes and host genes has only been reported in resistant and susceptible families of Chinese tongue sole infected with *V. harveyi* (3). Here, 207 significant interactions between the differential intestinal microbes and host DEGs were detected in resistant and susceptible individuals of Chinese tongue sole after *V. harveyi* infection by the combined analysis of RNA-seq and 16S rRNA, such as strong positive correlations between *Vibrio* and *IL17C*, *Vibrio* and *IL1B*, *Stenotrophomonas* and *H2-Ab1*, *Stenotrophomonas* and *H2-DMb1*, *Stenotrophomonas* and *IL34*, *Stenotrophomonas* and *CD74*, and strong negative correlations between *Vibrio* and *EDA*, *Vibrio* and *ITGA6*, *Vibrio* and *IL21*, *Stenotrophomonas* and *TLR5*, *Stenotrophomonas* and *C3*. Similarly, through the integrated analysis of metagenomic and transcriptomic, 116 strong host gene-microbe associations specific to resistant and susceptible families were identified in the intestine of Chinese tongue sole after infection with *V. harveyi* (3). In addition, the significant associations between the intestinal microbial community and the expression levels of *TNFα* and *IL1B* were also detected in the intestine of ayu infected with *V. anguillarum* (18),. Crucially, the genes involved in these correlations, such as *IL1B*, *IL17C*, *IL21*, *IL34*, *H2-Ab1*, *H2-DMb1*, *CD74*, *TLR5*, and *C3*, play crucial roles in regulating host innate and adaptive immune responses, and inflammatory reaction (74–82). These results suggested that these DEGs may interact with the intestinal microbes to regulate the vibriosis resistance of the host. Of course, it should be pointed out that our study has certain limitations. In details, we reported the potential roles of host gene-microbe interactions in the vibriosis of Chinese tongue sole. However, our study focused solely on correlations, and did not infer causal relationships between microbes and host genes, because establishing causality of gene-microbe interactions in fish species poses highly challenges. Therefore, future work employing *in vivo* or *in vitro* models may help to validate specific host gene-microbe correlations, elucidate their interaction mechanisms, and determine the directionality of their interactions.

## 5 Conclusion

In conclusion, this study indicated that *V. harveyi* infection caused significant differences in intestinal histopathology, intestinal microbial community composition, and host transcriptional expression between resistant and susceptible individuals of Chinese tongue sole with the same genetic background through integrating histopathology, microbiome profiling, and transcriptomic analysis. 16S rRNA sequencing indicated that *Vibrio* increased but *Stenotrophomonas*, *Chryseobacterium*, *Delftia*, and *Salinivibrio* decreased in the susceptible group. Besides, more DEGs (MHC II gene family members like *HLA-DPA1*, *H2-DMb1*, *H2-Aa*, *H2-Ab1*, *H2-Eb1*, *LOC103398979* and *HLA-DRA*) significantly enriched in immune-related GO terms and pathways were identified in the susceptible group. Furthermore, 207 strong gene-microbe correlations were detected through an integrative analysis of the differential intestinal microbes and host DEGs, but the causality between them has not yet been determined. Future research will focus on validating these potential biomarkers and gene-microbe correlations and elucidating the causal mechanisms of these host-microbe interactions, which will provide theoretical basis and candidate targets for genetic improvement of resistance to vibriosis in Chinese tongue sole.

## Data Availability

The datasets presented in this study can be found in online repositories. The names of the repository/repositories and accession number(s) can be found in the article/**Supplementary Material**.
