# Correction: Interactions between the intestinal microbiome and host genes in regulating vibriosis resistance in *Cynoglossus semilaevis*

**DOI:** 10.3389/fimmu.2026.1791642

**Published:** 2026-01-28

**Authors:** Weiwei Zheng, Yadong Chen, Tao Yang, Zhihong Liu, Dong Xu, Huizong Han, Yaning Wang, Xiaoqing Xi, Tengteng Wang, Songlin Chen

**Affiliations:** 1 State Key Laboratory of Mariculture Biobreeding and Sustainable Goods, Yellow Sea Fisheries Research Institute, Chinese Academy of Fishery Sciences, Qingdao, Shandong, China; 2 Shandong Marine Resource and Environment Research Institute, Yantai, Shandong, China; 3 Rongcheng Marine Economic Development Center, Weihai, Shandong, China; 4 Laboratory for Marine Fisheries Science and Food Production Processes, Qingdao Marine Science and Technology Center, Qingdao, Shandong, China

**Keywords:** *Cynoglossus semilaevis*, intestinal microbiome, host genes, interactions, vibriosis resistance

The figures were in the wrong order. The current **Figure 3** should be **Figure 4**, the current **Figure 4** should be **Figure 10** and the current **Figure 10** should be **Figure 3**. The order has now been corrected.

The original version of this article has been updated.

