# Supplementary figures and images for: Interactions between the intestinal microbiome and host genes in regulating vibriosis resistance in Cynoglossus semilaevis
Source: Front Immunol. 2025 Sep 3;16:1644885. doi: 10.3389/fimmu.2025.1644885 (PMC12440730; doi:10.3389/fimmu.2025.1644885)

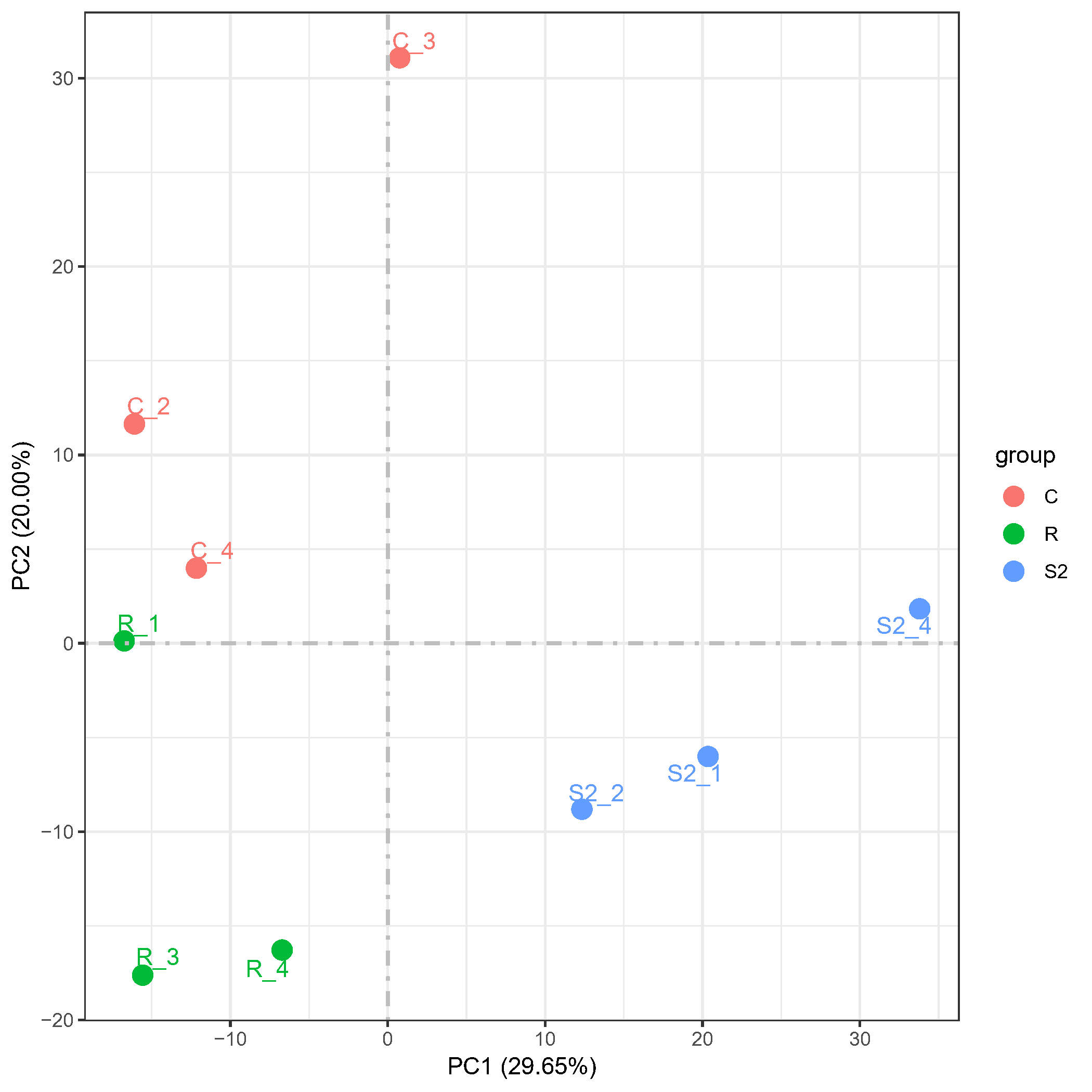

Supplement: Supplementary file 1 [file Image1.jpeg]
